# Substrains of Inbred Mice Differ in Their Physical Activity as a Behavior

**DOI:** 10.1155/2013/237260

**Published:** 2013-03-06

**Authors:** Dario Coletti, Emanuele Berardi, Paola Aulino, Eleonora Rossi, Viviana Moresi, Zhenlin Li, Sergio Adamo

**Affiliations:** ^1^UR4 Aging, Stress, Inflammation, University Pierre et Marie Curie Paris 6, 7 Quai Saint Bernard, 75005 Paris, France; ^2^Department of Anatomical, Histological, Forensic & Orthopaedic Sciences, Section of Histology & Medical Embryology, Sapienza University of Rome, Via Scarpa 16, 00161 Rome, Italy; ^3^Interuniversity Institute of Myology, 00161 Rome, Italy; ^4^Laboratory of Translational Cardiomyology, Department of Development and Regeneration, Katholieke Universiteit Leuven, 3000 Leuven, Belgium

## Abstract

Recent studies strengthen the belief that physical activity as a behavior has a genetic basis. Screening wheel-running behavior in inbred mouse strains highlighted differences among strains, showing that even very limited genetic differences deeply affect mouse behavior. We extended this observation to substrains of the same inbred mouse strain, that is, BALB/c mice. We found that only a minority of the population of one of these substrains, the BALB/c J, performs spontaneous physical activity. In addition, the runners of this substrain cover a significantly smaller distance than the average runners of two other substrains, namely, the BALB/c ByJ and the BALB/c AnNCrl. The latter shows a striking level of voluntary activity, with the average distance run/day reaching up to about 12 kilometers. These runners are not outstanders, but they represent the majority of the population, with important scientific and economic fallouts to be taken into account during experimental planning. Spontaneous activity persists in pathological conditions, such as cancer-associated cachexia. This important amount of physical activity results in a minor muscle adaptation to endurance exercise over a three-week period; indeed, only a nonsignificant increase in NADH transferase+ fibers occurs in this time frame.

## 1. Introduction

Exercise adaptations result from a coordinated response of multiple organ systems, including cardiovascular, pulmonary, endocrine-metabolic, immunologic, and skeletal muscle, recently reviewed by Boveris and Navarro [1], by Freidenreich and Volek [2], and by Perrino et al. [3]. Exercise training has been suggested as a promising countermeasure to prevent several disease states and as a rehabilitation tool aimed to restore both muscle strength and endurance, depending on the type of exercise [4]. Regular resistance exercise combined with adequate protein intake to maintain muscle mass is proposed to counteract sarcopenic obesity in an aging global population, a major public health challenge [5]. For all the above, rodent models of caloric intake and exercise are widely used [6] and novel molecular mechanisms underlying the effects of physical activity have been recently brought to light [7, 8]. Nonetheless, the anatomy and physiology of rodents differ significantly from those of humans. While it appears clear that *Homo sapiens* has evolved to support the svelte phenotype of an endurance runner [9], a better understanding of similarities and differences between human and animal models is becoming of paramount importance for translating discoveries in preclinical models to clinical settings.

The two main types of contractile activity that are classified as low muscular tension development over an extended duration, or high-tension generation of limited duration, are characteristic of endurance and resistance exercise, respectively. The aforementioned adaptive responses at the whole body and cellular and molecular levels depend on the mode of exercise performed [10]. For instance, increased strength [11–13], power [14], muscle cross-sectional area [15–17], RNA, and protein content [18] typically occur following resistance exercise training. Aerobic, endurance exercise training has been shown to enhance exercise capacity [19], augment maximal oxygen consumption [20], increase oxidative enzymes [21], and elevate mitochondrial content [22].

Several protocols of exercise training were developed for rodent models to mimic either resistance or endurance exercise. For instance, to climb a vertical ladder as a mode of progressive resistance exercise has been used for rats [23]. Recently, a very interesting equipment and system of resistance exercise, based on squat-type exercise for rodents, with control of training variables, has been validated [24]. The latter is based on a conditioning system composed of sound, light, and feeding devices, thus being not necessary to impose fasting or electric shock for the animal to perform the task proposed. Endurance exercise is based on more standardized protocols, basically running. The intensity-controlled treadmill exercise represents a well-characterized model of endurance exercise [25]. Slope and velocity of treadmill can be regulated and the animals are hosted in an enclosed chamber with a shock grid for motivating mice to run. One of its major advantages is the possibility of increasing time-wise exercise intensity, thus allowing the researcher to submit rodents to specific training programs. One of the drawbacks of treadmill is the fact that it may induce stress in the mice due to environmental, nonphysiological conditions. On the contrary, spontaneous exercise is often the favored type of exercise for experimental purposes since it is physiologic: it is performed at will, mostly during the nighttime; it mimics natural behavior, such as intermittent locomotion, typical of wildtype rodents; finally, it has been shown that such a voluntary activity is repeatable and stable within individual mice [26]. Hosting the mice in wheel-equipped cages, in which they exercise at will, classically induces such a spontaneous physical activity. A drawback of this approach is a certain degree of inter and intrapopulation variability, which makes absolutely necessary to individually monitor running activity by tachometers.

Small genetic differences may have a great influence on behavioral phenotypes [27]. Thus, the genetic background of different substrains should be carefully chosen, equated, and considered in the interpretation of mutant behavioral phenotypes. To this purpose, Knab et al. assessed the repeatability of a commonly used maximal exercise endurance treadmill test as well as voluntary physical activity measured by wheel running in mice: they found no significant differences in exercise endurance between different cohorts of BALB/c J and DBA/2 J mice indicating strains overall generally test the same [26]. Both strains are inbred mice; that is, populations that are nearly identical to each other in genotype due to long inbreeding. The usual procedure is mating of brother-sister pairs for 20 generations, which will result in lines that are roughly 98% genetically identical. Indeed, inbred strains of animals are frequently used in laboratories for experiments where for reproducibility of conclusions, all the test animals should be as similar as possible.

BALB/c are an inbred strain of mice distributed globally and are among the most widely used inbred strains. The founding animals of the strain (the “Bagg albino”) were obtained by Halsey J. Bagg of Memorial Hospital, NY, from a mouse dealer in Ohio in 1913. By 1935, the animals were in the possession of Muller's student, George Davis Snell, who moved them to The Jackson Laboratory. This stock provided the basis of all the BALB/c substrains that are now in use around the world. BALB/c ByJ (Jackson mice, donated to Jackson labs by Bailey J., in 1974) was separated from the BALB/c J strain in 1935. BALB/c ByJ mice have the advantage of better reproductive performance and less aggressiveness than the BALB/c J substrain and pose many other differences with the J substrain. Between the fifties and seventies, a third substrain got separated from the above-mentioned first two substrains, that is, the J and the ByJ: the Charles River AnNCrl (to Andervont in 1935 to NIH in 1951 from Andervont at F72 to Charles River in 1974 from NIH).

The three BALB/c substrains have been kept separated over decades and could have diverged to such an extent to develop sufficient genetic differences to account for behavioral differences among substrains, while remaining homogeneous within the same population. Mice may significantly differ for what concerns their physical activity as a behavior, which is of pivotal importance for the reproducibility and significance of studies exploiting exercise models. These differences may appear easily accountable when dealing with animals of different sexes or strains. However, we wondered whether even very fine differences (such as those distinguishing murine substrains of a single inbred strain) are able to determine significant behavioral differences. For this reason, we compared the physical activity behavior of the AnNCrl, the ByJ, and the J BALB/c mice and found striking differences concerning their willingness to run when hosted in wheel-equipped cages. Our findings have important experimental consequences with relevant economical and scientific fallouts.

## 2. Materials and Methods

### 2.1. Mice

Mice were generously provided by Janvier (Le Genest Saint Isle, St Berthevin Cedex, France). Throughout the study we used 7-week-old BALB/c mice of the following substrains: AnNCrl, ByJ, and J. We used a total of 21, 12, and 12 female mice of the substrains AnNCrl, ByJ, and J, respectively. Mice were allowed to settle in the animal facility for one day and then transferred to wheel equipped cages. Cachexia was induced by subcutaneous grafting, using a trocar of a 0.5 mm^3^ fragment of colon carcinoma (C26, obtained from the National Cancer Institute) in the dorsal region as previously described [28]. Mice were hosted in standard conditions with day/night cycles of 12 hours and food* ad libitum*. Mice were treated in strict accordance to the guidelines of the Institutional Animal Care and Use Committee and to national and European legislation, throughout the experiments.

### 2.2. Cages

Cages were purchased from Animal Care System (Centennal, CO). The wheels, structured as a circular open ladder with a diameter of 15 cm, were purchased as wheels for rodents in general customer pet shops. The tachometers, either model DC-4 or DC-9, were purchased from Decathlon. Readings were recorded every morning, before 10 am. Sporadic events of day running activity were observed. A Kleenex was introduced into the cage as material for the construction of a nest, with the aim to reduce stress due to being isolated (one animal per cage).

### 2.3. Tissue Immunohistochemical Analysis

NADH transferase staining was performed as described previously [28]. Morphometric analysis was performed on type IIB (low NADH transferase activity, glycolytic), type IIA/X (medium NADH transferase activity, intermediate), and type I (high NADH transferase activity, oxidative) fibers separately. For each muscle, the whole muscle cross-section was analyzed to calculate the percentage of each fiber type by using ImageJ 1.41 (freeware developed by Dr. W. Rasband at NIH, and available at http://rsb.info.nih.gov/ij/). The fibers with a medium and a high content in mitochondria were pooled and collectively considered as NADH transferase+ fibers, that is, oxidative. Photomicrographs were obtained by means of an Axioskop 2 plus system (Zeiss, Oberkochen, GE) or a Leica Leitz DMRB microscope fitted with a DFC300FX camera (Leica, Wetzlar, Germany).

### 2.4. Statistical Analysis

One-way or two-way analysis of variance (ANOVA) was used for one or two variate analysis, respectively. Either the Tukey LSD test or Student's *t*-test was used for the *post hoc* comparisons between specific groups, as indicated. The significance levels for these tests were set at a *P* < 0.05 or *P* < 0.01, as specified. Point and interval were estimated at the 95% confidence level. Statistical analyses were performed by using VassarStats, the website for statistical computation freely available at http://vassarstats.net/. 

## 3. Results

**Figure 1:**
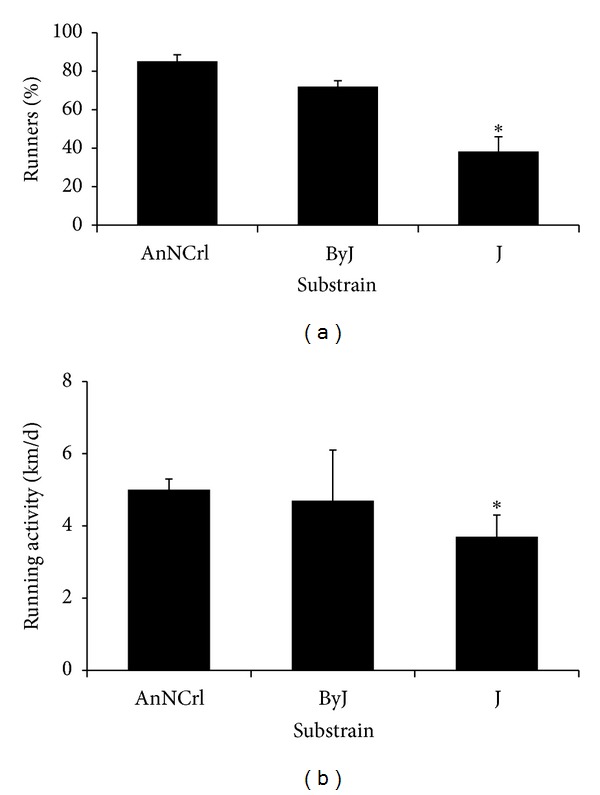
Different mouse substrains show differential physical activity as a behavior. Seven-week-old female BALB/c mice, belonging to three different substrains as indicated, were individually placed in wheel equipped cages. The running behavior (a) and the daily distance covered (b) were recorded for four days. The average ± SEM of at least three independent experiments, each one performed at least in quadruplicate, is shown. BALB/c J mice run significantly less than the other two substrains and the majority of this population do not show at all interest for wheel running. (a) *P < 0.01 by Tukey HSD test versus AnNCrl or versus ByJ. (b) *P < 0.05 by Sutdent's *t*-test versus AnNCrl.

Mice of three BALB/c substrains, that is, the AnNCrl, the ByJ, and the J, were obtained by the same vendor and hosted at the same time in the same animal facility. Each wheel-equipped cage was used for a single mouse, whose spontaneous wheel running activity was recorded by a commercial tachometer. The distance run over a period of four days was recorded daily; the average Km/day over such period of time was considered to minimize daily variations. Mice clearly divide into two populations of runners and nonrunners, the latter totally ignoring the wheel as such and showing no interest in wheel running at all. The threshold to define a runner was set to 1 Km of distance run on the wheel over the 4-day period of time. Several rounds of independent experiments, involving 6 mice for each of the three substrains, were repeated and the percentage of runners for each substrain in each experiment was assessed. In this way, an average percentage of runners in a given experiment and its associated SEM were calculated as a function of the substrain. We found that the runners were 85.1 ± 3.4%, 72.0 ± 3.0%, and 38.2 ± 7.7% of the AnNCrl, the ByJ, and the J, respectively (Figure 1(a)). The 95% confidence intervals were found to be 76.9–93.2%, 59.1–84.9%, and 18.5–57%, respectively. One-way ANOVA (*F* = 21.61; *P* < 0.0001) demonstrated a significant dependence of the percentage of runners on the substrain, with the BALB/c J running showing a significantly lower number of runners as compared to the other two substrains by Tukey's HSD *post hoc* test. Considering only the population of runners, we then assessed the distance run daily on the wheel by representatives of the three substrains. We found that the mice run 5.0 ± 0.3 Km/d, 4.7 ± 1.4 Km/d, and 3.7 ± 0.6 Km/d for the AnNCrl, the ByJ, and the J substrain, respectively (Figure 1(b)). The 95% confidence intervals were found to be 4.1–5.8 Km/d, 3.3–6.1 Km/d, and 2.2–5.0 Km/d, respectively. While one-way ANOVA (*F* = 1.88; *P* = 0.167) failed to demonstrate a dependence of the observed trend in the daily run distance on the basis of the substrain, we tentatively used the Student's *t*-test to statistically explore the difference shown by the J substrain and found that is significantly lower as compared to the AnNCrl substrain. In summary, we observed that the majority of the BALB/c J mice do not spontaneously run on a wheel, differently from the BALB/c AnNCrl and the BALB/c ByJ, the vast majority of which is willing to run. Moreover, even when the BALB/c J do run, they cover on average a smaller distance as compared to the BALB/c AnNCrl and the BALB/c ByJ mice. We concluded that the BALB/c AnNCrl is the best substrain for studies involving spontaneous physical activity, such as wheel running. For this reason, this substrain has been used throughout the rest of the study. 

**Figure 2:**
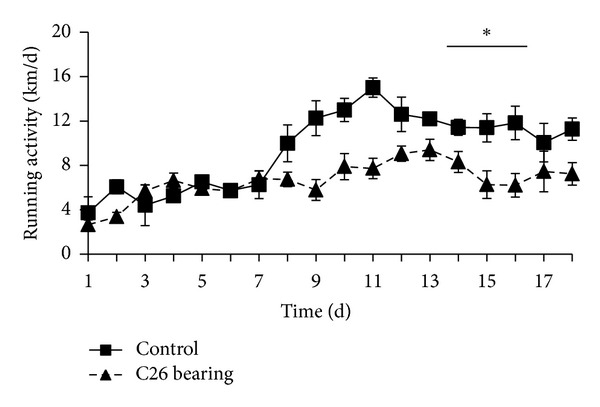
Kinetics of physical activity in healthy and C26-bearing BALB/c AnNCrl mice. Seven-week-old female BALB/c AnNCrl mice were individually placed in wheel equipped cages. At the same time a group (C26 bearing) was subcutaneously transplanted with the C26 colon carcinoma to induce muscle wasting. The running distance was daily recorded and averaged among replicates from at least three independent experiments. Both healthy and diseased mice do exercise, even though C26-bearing mice run for up to 6 Km/day, while healthy mice increased the daily distance run on the wheel to 11 Km/day. *P < 0.01 by Tukey's HSD test versus C26-bearing mice.

With the aim to assess whether the observed wheel running activity displayed features of exercise training and whether the 5 Km/day represented the upper limit of physical activity for BALB/c mice, we recorded the kinetics of mouse wheel running over almost three weeks. For this set of experiments, we decided to use the AnNCrl substrain of BALB/c mice, since these behaved as the most active mice. We remarked that mouse running behavior is biphasic, with a first week spent to familiarize with the wheel, with an outstanding (for the size of the animals), yet moderate, daily distance if compared to the second and third week of activity, in which the daily distance covered by the mice reaches a plateau that it is more than twice the initial Km/d (corresponding to more than 11 Km/days, Figure 2). In this context, we also wondered whether mice were able to perform voluntary physical activity in pathological conditions, such as cancer-induced cachexia [29]. Thus, we recorded the daily running activity of C26 colon carcinoma-bearing mice, which develop a progressive and severe form of muscle wasting associated to weakness and fatigue [28]. C26-bearing mice run about the same Km/d as controls in the first week of activity, when the tumor size is still negligible; however, they do not show the same progressive increase in the distance run on the wheel as the controls in the second week of activity. With the disease progression and overt cachexia, they keep running for about 7 Km/d, which is a striking amount of exercise, considering that tumor-bearing mice lose about 25% of the body weight in three weeks. Two-way ANOVA calculated over the last four days of the recordings (i.e., from day 15 to day 18) shows that only the presence of the tumor significantly affects the running behavior, with no interference with time (*F* = 19.74; *P* < 0.0001; Tukey's HSD *P* < 0.01 for control versus C26 bearing). These data indicate that the BALB/c AnNCrl mice not only spontaneously run several Km per day but also have the tendency to progressively increase the distance covered on the wheel. Mice bearing a tumor, a condition which deeply affects muscle mass and function, are still capable of performing a significant amount of physical activity, albeit to a lesser extent than healthy mice. This indicated that wheel running could be exploited as a model for endurance exercise intervention in pathological settings in rodents.

**Figure 3:**
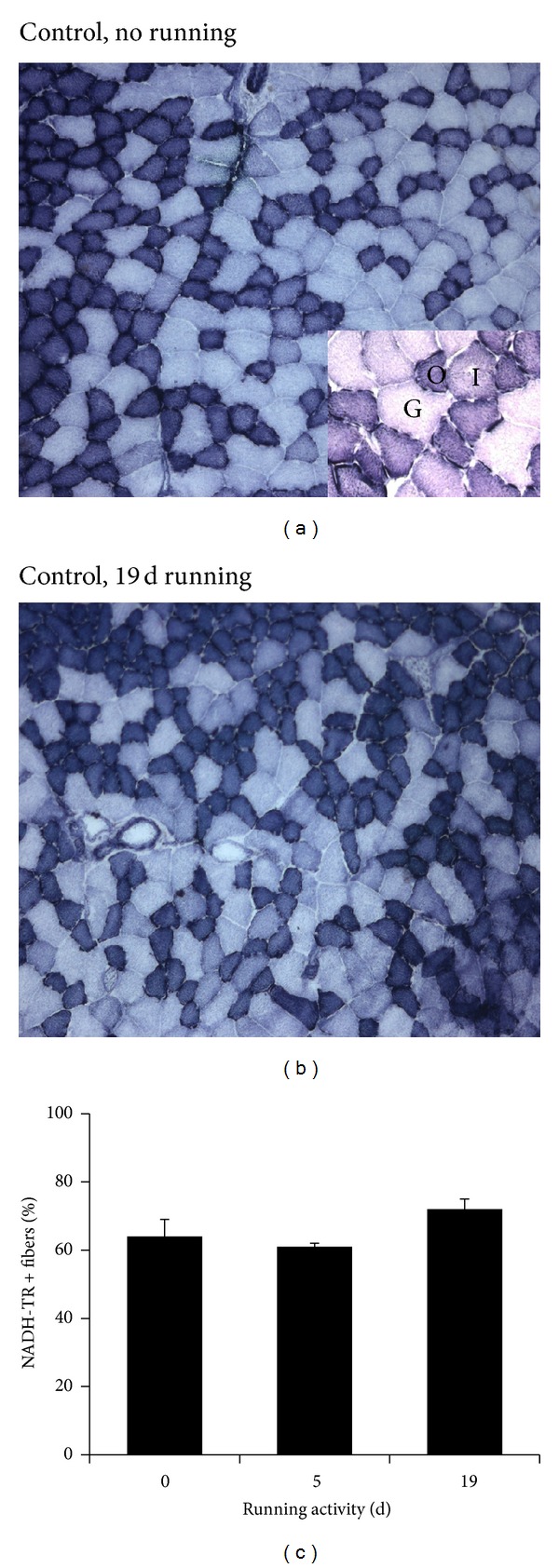
Muscle metabolic adaptations to wheel running in BALB/c AnNCrl mice. Histochemistry for NADH transferase activity highlights oxidative (O), intermediate (I), and glycolytic (G) fibers; that is, muscle fibers that are rich (O or I) and poor in mitochondrial content, respectively. The inset at higher magnification allows to differentiate among the three types of fibers. O + I fibers were collectively considered as rich in NADH transferase (*NADH-TR  + fibers*). Representative photomicrographs of the TA of (a) control, nonexercised mouse and (b) a mouse following nineteen days of exercise. The percentage of NADH-TR+ fibers was quantified in replicate, after no (*0*), five (5), or nineteen (*19*) days of wheel running.

Endurance training activates metabolic pathways and remodeling in skeletal muscle. A prominent feature of this type of exercise is the stimulation of Krebs' cycle and the mitochondriogenesis in muscle fibers. Since in BALB/c AnNCrl mice we observed a spontaneous, yet significant, increase in physical activity during the second week of permanence in wheel-equipped cages, we hypothesized that the increased performance was associated to metabolic changes making the musculature adapted to endurance exercise. Thus, we performed a histochemical analysis of the skeletal muscle of BALB/c AnNCrl mice following two different periods of wheel running, that is, five and nineteen days, to monitor phenomena of fiber conversion to a more oxidative phenotype upon exercise. By NADH transferase staining on the *Tibialis anterior* (TA) muscle (Figures 3(a) and 3(b)), we highlighted the fibers rich in mitochondria (oxidative and intermediate fibers, typically corresponding to type I and IIA or X). The TA was chosen for its mixed population of fiber types (all types are represented), which appeared particularly suitable for studying shifts in fiber type. While we observed an increase in the number of NADH transferase+ fibers from 64 ± 5% to 72 ± 3% following nineteen days of exercise, one-way ANOVA (*F* = 2.39; *P* = 0.162) showed the lack of a statistically significant effect by exercise on this parameter (Figure 3(c)).

## 4. Discussion

We have shown that three substrains of the same inbred mice, namely, the BALB/c AnNCrl, ByJ, and J, display striking differences in their behavior concerning spontaneous physical activity. This phenomenon was observed in spite of the fact that the three substrains display remarkable genetic similarities, exemplified by the absence of histocompatibility barriers. The fact that the vast majority of the AnNCrl and, to a lesser extent, of the ByJ mice run for several Km per day distinguishes these two substrains from the J mice. To our knowledge, this is the first paper on significant behavioral differences among substrains of the same inbred mouse strain. Researchers planning to perform experiments requiring wheel running should be aware of these unexpected findings. In fact, the selection of the J substrain, which is less prone to wheel running, would determine a very little amount of exercise performed by a limited number of mice. Interestingly, the J substrain is the cheapest (at least with the vendor used for this study) however, if a relevant number of animals are excluded from a study since they do not exercise, the costs must be recalculated. Researchers that, for an experimental reason, need to use the J substrain and that absolutely require that they exercise by wheel running may consider selecting the mice in preliminary experiments according to their running behavior to sort the runners from the nonrunners in advance. Otherwise, researchers interested in using BALB/c mice for exercise-related experiments may simply want to choose either the AnNCrl or the ByJ substrain.

The idea that different mice strain behave differently about wheel running is not new, but, again, our study shows that even very fine differences (such as those distinguishing substrains) are able to determine significant behavioral differences. Several studies associated genetic influence with physical activity, but animal studies were often conducted with only one sex or a limited number of strains, thus reducing the genomic coverage and generality of the result that Lightfoot et al. clearly showed that physical activity as a behavior has a genetic basis [30]. Their results suggest that potential genetic mechanisms arising from traditional noncoding regions of the genome may be involved in regulation of physical activity [30]. Of course, other studies clearly show that mouse substrains differ for several features other than physical activity as a behavior. For instance, it has been shown that the genetic background of the four different mouse substrains affects their vulnerability to cope with environmental challenges, such as exposure to novelty; the authors consistently suggest considering substrain-specific guidelines and protocols, taking the substrain-specific adaptive capabilities into account [31]. We totally agree with the authors of this study. Another intriguing study showed that two substrains of BALB/c mice, the BALB/cByJ and the BALB/cAnNCr, are resistant and susceptible, respectively, to Theiler's murine encephalomyelitis, a virus-induced demyelinating disease [32]. The fact that the two substrains are histocompatible makes them a nice model for studying mechanisms of virus infection, since they permit the transfer of cells between naturally resistant and naturally susceptible mice in the absence of immunodepression. Similarly, one could foresee the possibility of satellite cell grafts among different BALB/c substrains to verify whether fine genetic differences are responsible for differential muscle stem cell features, such as their capability of being engrafted into regenerating muscles; one could even wonder whether satellite cells from spontaneous runners could donate to the derived muscle fibers intrinsic mechanical or contractile properties more suitable for running.

Our study is in agreement with previous results showing that mice spontaneously run for 5 to 10 Km per day [30]. This incredible level of voluntary activity is an important fact to keep in mind and represents an outstanding feat for such a diminutive species. It has been stated that “such distances covered daily by us much larger humans would probably cure most of the epidemic diseases facing the world, including obesity and type 2 diabetes” [33]. 

Running is associated with distinct metabolic adaptations of the skeletal muscle [34, 35]. In particular, it has been reported that voluntary running exercise induces a steady increase in the percentage of NADH transferase-positive fibers in the TA muscle, which was significant after 4 weeks of voluntary exercise [36]. We obtained very similar results in terms of exercise effect on the percentage of NADH transferase+ fibers, with the exception that we failed to demonstrate the significancy of such an effect. This may be due to several differences between the two studies: the mouse strain (BALB/c, C57/Bl6), the sex (female, male), the distance (5 Km/day, 6.8 Km/day on average), and the time frame (3 weeks, 4 weeks); each of these differences could be sufficient to explain why we could not observe a statistically significant increase in the oxidative fibers of exercise mice. The observed trend is consistent with an increased demand of muscle oxidative capacity suggesting that endurance exercise invariably affects muscle metabolism by favoring the oxidative muscle fiber phenotype.

Finally, it should be noted that the therapeutics and ergogenic effects of controlled exercised as opposed to spontaneous exercise may differ significantly in rodents. Intriguingly, the two types of exercise may have very different outputs depending on the targeted organ. For example, voluntary activity causes a more evident plastic changes in the hippocampal formation of rat than that one induced by forced exercise [37]. 

## 5. Conclusion

Recognizing the proven benefits of exercise training on health outcomes and the trend towards increasing inactivity at the population level has made recommending exercise a directive of paramount importance. In parallel, studies on organismal and muscle-specific adaptations to increased physical activity steadily increase over time, as shown by the trend in PubMed citations with the keywords exercise and endurance/resistance. With such a proliferation of animal and experimental models dedicated to exercise, it is important to clearly define the major features of the experimental models used in a given study and to be very formal in assessing to which extent generalization of the results can be driven. We report here that three substrains of the same inbred mouse strain, the BALB/c, display significant differences in physical activity as a behavior. We propose that not only the strain of mice used but also the substrain must be clearly specified and chosen consciously, since the differences in spontaneous physical activity between substrains can impact exercise-induced muscle adaptations.
